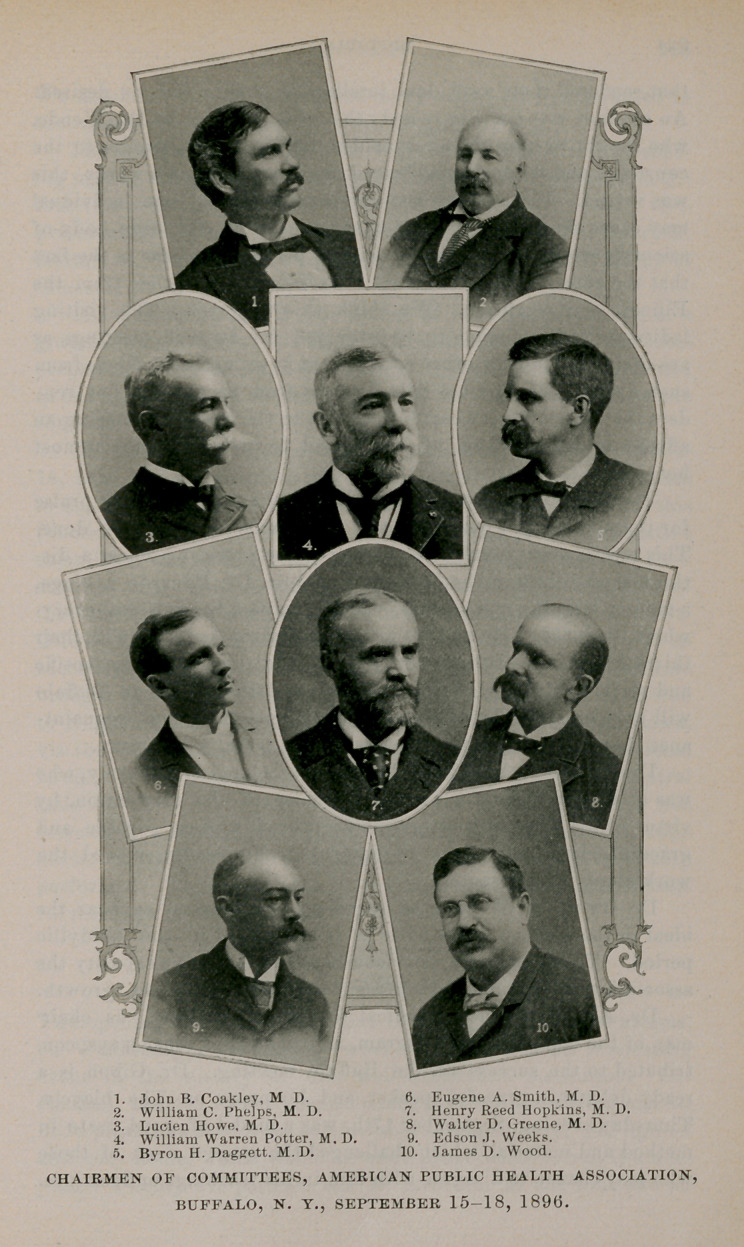# American Public Health Association

**Published:** 1896-10

**Authors:** 


A Monthly Review of Medicine and Surgery.

*EDITORS:*

THOMAS LOTHROP, M. D. - - WM. WARREN POTTER, M. D.

All communications, whether of a literary or business nature, should be addressed to the managing editor: 284 Franklin Street, Buffalo, N. Y.

Vol. XXXVI. OCTOBER, 1896. No. 3.

### AMERICAN PUBLIC HEALTH ASSOCIATION.

According to the announcements in previous issues of the Journal, this important organisation held its twenty-fourth annual meeting in Buffalo, September 15–18, 1896. The history and purposes of the association were given in our September issue, but it remains for us to speak at this time especially of the recent meeting.

In the first place, the season for the gathering was well chosen. Buffalo is at its best in summer and early autumn, and the weather could scarcely have been better than during the week of the meeting. Again, the place in which it was held was a fortunate selection. Ellicott square furnishes more conveniences for such a meeting than can be found under one roof in Buffalo, or, for that matter, we believe, in any other city within the jurisdiction of the association. Furthermore, the arrangements were completed with an unusual degree of perfection, thanks to the leadership of Dr. Ernest Wende, chairman of the local committee. The frequent meetings of the executive committee during the summer months led to concerted action on the part of the several sub-committees, so that each had its work well in hand.

In justice to truth it must be said, however, that one or two criticisms at this point are pertinent. First, and perhaps foremost, stands out the fact that the acoustic properties of the hall in which the scientific work of the association was done were the very worst. This was accentuated all the more by the noise from street traffic, which latter made it necessary to close the windows on the South Division street side. Hence, the members were compelled to choose between a stifling atmosphere or an exasperating noise

Ernest Wende

HEALTH COMMISSIONER OF BUFFALO, CHAIRMAN EXECUTIVE COMMITTEE AMERICAN PUBLIC HEALTH ASSOCIATION, BUFFALO MEETING, SEPTEMBER 15-18, 1896.

1. John B. Coakley, M D.
2. William C. Phelps, M. D.
3. Lucien Howe, M. D.
4. William Warren Potter, M. D.
5. Byron H. Daggett. M. D.
6. Eugene A. Smith, M. D.
7. Henry Reed Hopkins, M. D.
8. Walter D. Greene, M. D.
9. Edson J. Weeks.
10. James D. Wood.

CHAIRMEN OF COMMITTEES, AMERICAN PUBLIC HEALTH ASSOCIATION,

BUFFALO, N. Y., SEPTEMBER 15–18, 1896.

that rendered their work less intelligible than could be desired. An attempt was made to remedy the noise nuisance by Dr. Wende, who sought to have the street laid in tanbark ; but, thanks to the courteous and learned president of the board of public works, this was vetoed. It is strange how the perverseness of one individual may cause the discomfort, not to say confusion, of a large body of scientific workers. Another criticism pertinent to offer is the fact that the entertainment committee excluded the ladies from the Ellicott club reception. We think this an error. The visiting ladies are quite as much to be provided for at such meetings as are their husbands or other relatives, and to exclude them from such a social function not only detracts from its pleasure but renders the entertainment committee open to the charge of causing an affront to guests whom all are bound to treat with the utmost hospitality.

Turning now to the meeting itself, we have nothing but praise for the number and quality of the attendance or the work done. This meeting was notable for having as its presiding officer a distinguished citizen of the Mexican Republic, Dr. Eduardo Licéaga, a man of learning and science, whose dignified bearing was everywhere observed. He and his accomplished son, together with their thirty Mexican confréres, were decided additions to the scientific and social qualities of the association, and their visit to Buffalo will long be remembered by many of its citizens whose acquaintance they made and whose friendship, we trust, will endure.

Dr. Alfred A. Woodhull, Lieutenant-Colonel U. S. Army, who was on this occasion the executive officer of the association, by virtue of his office as vice-president, proved a most capable and graceful chairman, whose parliamentary knowledge moved the work along with despatch.

Dr. Irving A. Watson, as secretary, approaches as near the ideal in the conduct of his office as is permitted in this unidyllic period. To his sagacity, courteousness and executive capacity the association owes much of its present influence and rapid growth.

Dr. Albert L. Gihon, medical director U. S. Navy, as chairman of the committee on program, and in various other ways, contributed to the success of the Buffalo meeting. Dr. Gihon is a ready debater, a graceful speaker, and his paper on The bicycle, Thursday evening, September 17th, was a model of good taste in method and material, which challenged the respect even of those who may chance to disagree with him in his teachings or doctrines.

We should be pleased to speak of many others who contributed to the success of the Buffalo meeting did space permit.

The association chose the following-named officers for the ensuing year: President, Dr. Henry B. Horlbeck, of Charleston, S. C.; first vice-president, Dr. Peter H. Bryce, of Toronto; second vice-president, Dr. Ernest Wende, of Buffalo; treasurer, Dr. Henry D. Holton, of Brattleboro, Vt.; secretary, Dr. Irving A. Watson, of Concord, N. H.

The election of Dr. Wende as one of the vice-presidents is a graceful recognition of his successful administration of the local preparations for the Buffalo meeting. Philadelphia was selected as the place in which to meet next year, the date to be fixed by the council.

The association adjourned at 2 o'clock P. M., Friday, September 18, 1896, and the members and guests joined the local committee in an excursion around the harbor. An interesting event of this function, arranged by Dr. E. C. W. O'Brien, of the local executive committee, was an exhibition of maneuvers by the life-saving crew attached to the Buffalo station. All seemed to enjoy this part of the entertainment, and the thanks of the committee are due to Dr. O'Brien for his thoughtfulness in proposing and carrying out so successfully this part of the program. Finally, the exercises were brought to a close on Saturday, September 19th, by an excursion to Niagara Falls, via Chippewa, thence by trolley to Queenstown, via the Gorge road to the Falls, and a dinner at the International hotel. The tourists returned to Buffalo via the New York Central railway. This part of the progıam, so far as transportation was concerned, was arranged by Mr. Edson J. Weeks, chairman committee on transportation and railroads, whose work was most ably performed. Thus ended one of the most satisfactory and successful meetings in the history of the association.

## Figures and Tables

**Figure f1:**
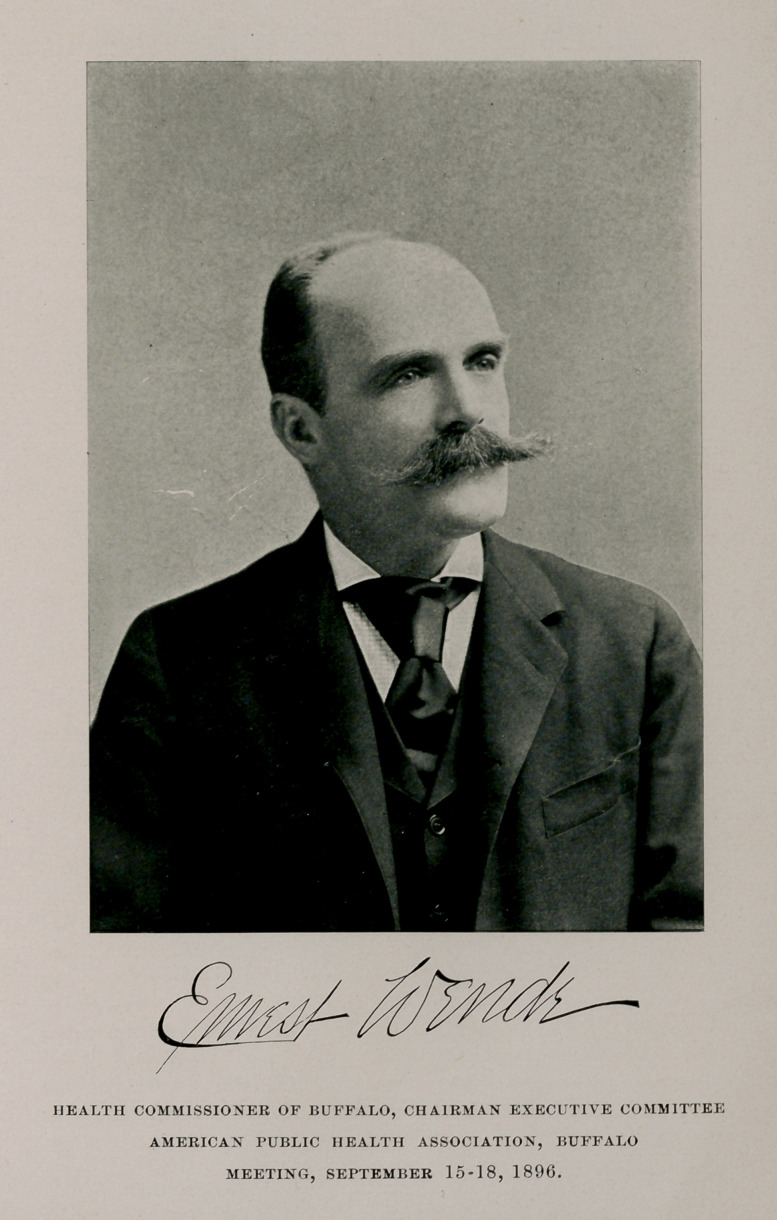


**Figure f2:**